# Trends of genetic changes uncovered by Env- and Eigen-GWAS in wheat and barley

**DOI:** 10.1007/s00122-021-03991-z

**Published:** 2021-11-15

**Authors:** Rajiv Sharma, James Cockram, Keith A. Gardner, Joanne Russell, Luke Ramsay, William T. B. Thomas, Donal M. O’Sullivan, Wayne Powell, Ian J. Mackay

**Affiliations:** 1 Scotland’s Rural College (SRUC), Kings Buildings, West Mains Road, Edinburgh, EH9 3JG UK; 2 The John Bingham Laboratory, NIAB, 93 Lawrence Weaver Road, Cambridge, CB3 0LE UK; 3 The James Hutton Institute, Invergowrie, Dundee, DD2 5DA UK; 4 School of Agriculture, Policy and Development, University of Reading, Reading, RG6 6AR UK

## Abstract

**Key message:**

Variety age and population structure detect novel QTL for yield and adaptation in wheat and barley without the need to phenotype.

**Abstract:**

The process of crop breeding over the last century has delivered new varieties with increased genetic gains, resulting in higher crop performance and yield. However, in many cases, the alleles and genomic regions underpinning this success remain unknown. This is partly due to the difficulty of generating sufficient phenotypic data on large numbers of historical varieties to enable such analyses. Here we demonstrate the ability to circumvent such bottlenecks by identifying genomic regions selected over 100 years of crop breeding using age of a variety as a surrogate for yield. Rather than collecting phenotype data, we deployed ‘environmental genome-wide association scans’ (EnvGWAS) based on variety age in two of the world’s most important crops, wheat and barley, and detected strong signals of selection across both genomes. EnvGWAS identified 16 genomic regions in barley and 10 in wheat with contrasting patterns between spring and winter types of the two crops. To further examine changes in genome structure, we used the genomic relationship matrix of the genotypic data to derive eigenvectors for analysis in EigenGWAS. This detected seven major chromosomal introgressions that contributed to adaptation in wheat. EigenGWAS and EnvGWAS based on variety age avoid costly phenotyping and facilitate the identification of genomic tracts that have been under selection during breeding. Our results demonstrate the potential of using historical cultivar collections coupled with genomic data to identify chromosomal regions under selection and may help guide future plant breeding strategies to maximise the rate of genetic gain and adaptation.

**Supplementary Information:**

The online version contains supplementary material available at 10.1007/s00122-021-03991-z.

## Introduction

In the last century, significant improvements in yield and quality have been reported in almost all crop species as a result of plant breeding driven by market demand (Fischer and Edmeades 2010). However, the growing demand for food, feed and fibre to meet the expanding global human population requires an acceleration in the pace of crop genetic improvement (Varshney et al. 2018). Identification of the genetic loci responsible for these changes will help accelerate the genetic gains required to meet future food security needs, via their incorporation in marker-assisted selection breeding strategies (Chiurugwi et al. 2019). Over the last decade, genome-wide association studies (GWASs) have become a prominent method for genetic analysis in plants (Ingvarsson and Street 2011). In crops, GWAS require trait data on large collections of varieties or accessions, which are typically expensive to collect and can therefore result in underpowered studies with relatively low numbers of lines (Macarthur 2012; Mackay et al. 2019). An alternative is to exploit the availability of historical data, such as that collected during varietal development programmes.

For almost every major crop, yield is the most important breeding target. Breeding programmes invest large amounts of resources into realising the incremental genetic gains in yield that are required for continual varietal improvement. Accordingly, the process of developing new crop varieties involves rigorous screening in large multi-location and multi-environmental trials over several years. Large historical phenotypic data sets from such trials have been successfully employed for GWAS in the past (Huang and Han 2014) and in several cases have identified the functional genes underlying the genetic control of the investigated traits (Cockram et al. 2010; Hamblin et al. 2011; Ramsay et al. 2011; Comadran et al. 2012). However, the availability of seed for variety collections with appropriate trait data is not common for many crops. Alternatively, seed of historical varieties may be available, but the associated trait data may be lost or disjointed. In both cases, the cost of collecting de novo trait data can be prohibitive. In many cases however, the release date, subsequently termed here ‘age’, of varieties is known. Given that, in most crops, the breeding process has improved the genetic potential of key agronomic traits over time, variety age can be used as a surrogate measure of merit and mapped in GWAS. The approach in which environmental or any other non-genetic variables are treated as traits in GWAS to map loci associated with those variables, has been termed EnvGWAS (Li et al. 2019), and we also adopt that terminology for our analyses of variety age. However, for many crops, the predominant genetic change over time has been to increase yield (e.g. Mackay et al. 2011), and the age of a variety may function directly as a surrogate for yield, although loci detected may also be associated with other temporal changes. EnvGWAS on variety age can also be regarded as a simple genome-wide test for genetic loci under directional selection, which may be subsequently associated with traits. This approach may also provide a way of identifying alleles associated with adaptation (Rowan et al. 2020), which otherwise have been difficult to detect. Finally, EnvGWAS can be a cost-effective strategy since it can access large pre-existing datasets but is not dependent on historical or de novo trait data.

A related approach requiring no-trait data is EigenGWAS (Chen et al. 2016). Using genotypic data alone, the singular value decomposition of the genomic relationship matrix provides loadings (eigenvectors) for each variety on each eigenvalue of the matrix. For the largest eigenvalues, these loadings are then treated as independent traits for GWAS. Significant associations with any particular component highlight genomic regions or markers of greatest importance for that eigenvalue and therefore the potential major drivers of population structure. Subsequent study of varieties differing in these regions may also be interpretable in terms of drivers of adaptation. EigenGWAS and EnvGWAS have recently been used to study diversity among maize landraces and identify lines and traits suitable for downstream analysis without large-scale phenotyping (Li et al. 2019).

In this study, we demonstrate for the first time the utility of treating variety age as a surrogate trait for crop productivity when combined with EnvGWAS and EigenGWAS to identify target regions and quantitative trait loci (QTL) underpinning genetic improvements in crop performance that have occurred during modern plant breeding. This is a powerful but cost-effective method that does not require extensive trait data or complex software. We demonstrate the utility of these complementary approaches by: i) using EnvGWAS on variety age to identify loci responsible for genetic improvement in four complimentary datasets of modern winter and spring types of wheat (*Triticum aestivum*) and barley (*Hordeum vulgare*) from the United Kingdom (UK) and Brazil. (2) Validating the results from (1) by GWAS on subsets of these varieties for which historic yield data were also available. (3) Evaluating the temporal changes of allelic state at the loci identified. (4) Performing EigenGWAS on the same four datasets. EigenGWAS compliments EnvGWAS in that it too does not require trait data and may also identify genomic regions that have undergone selection. However, unlike EnvGWAS, it does not explicitly search for regions associated with variety age and is more likely to detect features associated with local adaptation, which may change little in frequency over time. As far as we are aware, no EnvGWAS analysis has been published in plants for which variety age has been used as a trait. The combination of EnvGWAS with EigenGWAS used here provides insights into the recent breeding history and population structure of two of the world’s most important crops and highlights the effectiveness and simplicity of these approaches to study recent selection history without the requirement for phenotype data.

## Materials and methods

### Genotyping

Genotypic data were sourced from NIAB (https://www.niab.com/research/agricultural-crop-research/resources) and JHI (http://www.barleyhub.org/projects/impromalt/) by permission through WAGTAIL and IMPROMALT projects.

For wheat, 14,654 SNPs derived from genotyping with the 90-K Illumina iSelect SNP array (Wang et al. 2014) generated within the Biotechnology and Biological Sciences Research Council grant BB/J002542/1 were sourced with permission from NIAB, and available at https://www.niab.com/research/agricultural-crop-research/resources. For barley, 43,799 SNPs genotyped using the 50-K Illumina iSelect array (Bayer et al. 2017) were sourced from (Looseley et al. 2020). Genetic maps for wheat (Wang et al. 2014) and barley (Bayer et al. 2017; Looseley et al. 2020) have been previously described. The physical map locations of wheat and barley SNPs were retrieved from Sun et al. 2020 and Bayer et al. 2017, respectively. SNPs with a minor allele frequency < 5%, missing values > 10% and heterozygosity > 10% were removed, leaving 12,656 wheat SNPs and 25,562 barley SNPs for downstream analyses.

### EnvGWAS and EigenGWAS analyses

EnvGWAS and EigenGWAS analyses were performed using the R-package GWASpoly (Rosyara et al. 2016) implemented in R version 3.5.2 (http://www.R-project.org/). To determine the population structure of the panels, principal component analysis (PCA) was performed using the R-package SNPRelate (Zheng et al. 2012). The SNP-trait association analyses were conducted using a linear mixed model designated the K-model (kinship) by Yu et al. (2006). In summary, the linear mixed model is described as follows:

$$y = Xb + Zg + \varepsilon$$

where $y$ indicates the phenotypic vector for varieties (year of entry into trial in EnvGWAS, one of the first 10 principal component (PC) in EigenGWAS and yield in GWAS); $b$ is a vector of fixed effects, here a mean effect and an effect for a single SNP; $X$ is the design matrix for the fixed effects; here a vector of 1’s for the mean and a vector of zeros and 1 s indicating the presence or absence of the reference allele in the inbred lines; *g* models the genetic background of each line as a vector of random effects with mean zero and variance *σ*_*g*_^2^. $Z$ is the incidence matrix for the residual genetic effects, assigning varieties to observations. Random residual effects are in the vector $\varepsilon$ with mean 0 and variance *σ*_*e*_^2^.

Effects are estimated as:

$$\left[ {\widehat{{\begin{array}{*{20}c} b \\ {\hat{g}} \\ \end{array} }}} \right] = \left[ {\begin{array}{*{20}c} {X^{\prime}X } & { X^{\prime}Z} \\ {Z^{\prime}X} & { Z^{^{\prime}} Z + G^{ - 1} } \\ \end{array} } \right]^{ - 1} \left[ {\begin{array}{*{20}c} {X^{\prime}y} \\ {Z^{\prime}y} \\ \end{array} } \right]$$

where *G* = *K*σ_g_^2^ is a square matrix with elements of *K* estimated by van Raden’s (2008) method as:

$$k_{ij} = \, \Sigma \, \left[ {\left( {w_{ik} - 2p_{k} } \right)\left( {w_{jk} - 2p_{k} } \right)} \right]/2\Sigma \, p_{k} q_{k}$$

where *w*_*ij*_ (_*jk*_) is equal to the standardised marker score for marker k in variety *i* (*j*), *p*_*k*_ is the average allele frequency of marker k and *q*_*k*_ = 1 − *p*_*k*_. Summation is over markers.

A subset of markers pruned on genetic map distance was used to estimate *G* (741 for wheat and 2500 for barley). Marker coverage is variable over the genome, and the pruned set of SNPs better represent whole genome level relationships among varieties. Pruning was based on genetic positions using TASSEL 5.0 (Bradbury et al. 2007) to a minimum 5 cM between adjacent markers. Although the barley genome is substantially smaller than the wheat genome, more markers remained after pruning; a consequence of denser initial coverage and the uneven distribution of markers in wheat with marker clusters associated with introgressions and marker deserts in the D genome. Given that wheat and barley are highly self-pollinated species, an additive model is appropriate in the analysis with marker effects estimated as the effect of carrying the reference allele. All effects, variances and the relationship matrix *G* were estimated using GWASpoly.

Inclusion of the relationship matrix *G* subsumes genome-wide changes over time resulting from drift. This is true of GWAS on historical datasets for any trait.

For ease of comparison across GWAS scans, the threshold for significance was set to –log_10_ (*p*-value) = 4.0 which in our GWAS scans was above the threshold obtained using a false discovery rate of 5% (http://www.strimmerlab.org/software/fdrtool/index.html). GWAS was carried out on all markers, including those selected for estimation of kinship. Manhattan plots and circular plots were generated using R-packages qqman (Turner 2018) and CMplot (Yin et al. 2021), respectively.

### Germplasm, age and trait data

For both wheat and barley, we selected two panels of varieties representing national list entries and some older varieties from the UK (404 winter wheat; 297 winter and 406 spring barleys) and Brazil (355 spring wheat) (Supplementary Table S1). The Brazilian spring wheat panel included entries released between, 1922–2013. Year of varietal release and trait data were obtained from Mellers et al. 2020. The UK wheat panel consists of winter wheat varieties that were either registered or in use from 1916 to 2010. The winter and spring barley panels consisted of varieties grown in the UK from 1960 to 2016. Only two-rowed spike morphology types were included, and all hybrid varieties were excluded. Variety age for UK germplasm was determined from the year of entry into national list trials or from the first reported year of trial data and was manually checked across different local data and published sources ((Mackay et al. 2011); https://ahdb.org.uk/rl & https://www.gov.uk/government/publications/plant-varieties-and-seeds-gazette-2020https://www.niab.com/services/seed-certification/botanical-descriptions-varieties) with unresolvable ambiguities removed, reducing the UK wheat panel from 450 to 404 varieties. Following Mackay et al. 2011, only varieties with either three-year trials data or equivalently which were known to be successful in national list trials were included in the dataset. In addition to variety age, we computed lifespan of UK varieties as the difference between the last and first years in national trials plus one. This is usually equally to the total number of years each variety remained in trial, though with some rare breaks in the testing sequence over years. Grain yield data for the UK wheat and barley panels were sourced from (Mackay et al. 2011), previously modelled with REML, fitted in GenStat10 (Payne et al. 2007) as:

$$y_{ijk} = \mu + v_{i} + s_{j} + vs_{ij} + l_{jk} + e_{ijk}$$

where *y*_*ijk*_ is the varietal yield data of variety *i* in year *j* at location *k*; the trial series mean is denoted as *µ*; the effect of the *i*th variety is represented as *v*_*i*_; year effect of the *j*th year is represented as *s*_*j*_; the interaction of variety *i* in year *j* is represented as *vs*_*ij*_; *l*_*jk*_ is the effect of location *k* within year *j*; and the residual is *e*_*ijk*_, accounting for the combined effects of within-trial error and variety x site within-year interaction. Location effects within year and the interaction of variety with year were treated as random effect and varieties and years as fixed effects. Further details are in Mackay et al. (2011).

#### MAGIC wheat analysis

Three highly significant genomic regions (− log10 (*p*) > 6.0) from the wheat EnvGWAS for age were tested for association with the 38 agronomic characteristics recorded in the ‘NIAB Diverse MAGIC' population (Scott et al. 2021). This population was created from sixteen distinct founders derived from historical UK bread wheat varieties released between 1935 and 2004 and was utilised here as an independent resource to detect direct trait effects for the highly important genetic areas found in the EnvGWAS for age.

Analysis was performed in R version 4.0.5 using adjustments for the funnel structure of the cross as given in Scott et al. (2021). Corresponding matching SNPs anchored to physical map positions were obtained which were interrogated for associations in MAGIC RILs. All data used were obtained from the following website that hosts the genotyping and phenotyping data of the 550 MAGIC-diverse RILs; http://mtweb.cs.ucl.ac.uk/mus/www/MAGICdiverse/index.html.

## Results

All markers were included in the GWAS, including those selected for estimation of kinship. However, dropping those markers from the association tests had no effect on the pattern of results. For simplicity, only results from the full set of markers are presented here.

### Year of variety release as a surrogate measure for yield

We have retrieved historical wheat and barley variety means from the analyses of (Mackay et al. 2011) wherein yield of varieties is adjusted for the effect of locations and years by fitting a linear mixed model using REML. The Pearson correlations between historical yield data and age of variety were calculated for the subsets of 192 UK wheat and 197 UK barley varieties for which historical yield data were available (Supplementary Fig. S1). High correlations between yield and year of release (0.896 and − 0.974) were found in both UK data sets. This confirms year of release could be used as a good measure of genetic progress in UK wheat and barley yield potential. No historical yield data for the Brazilian wheat panel were available.

### EnvGWAS for variety age

**Fig. 1:**
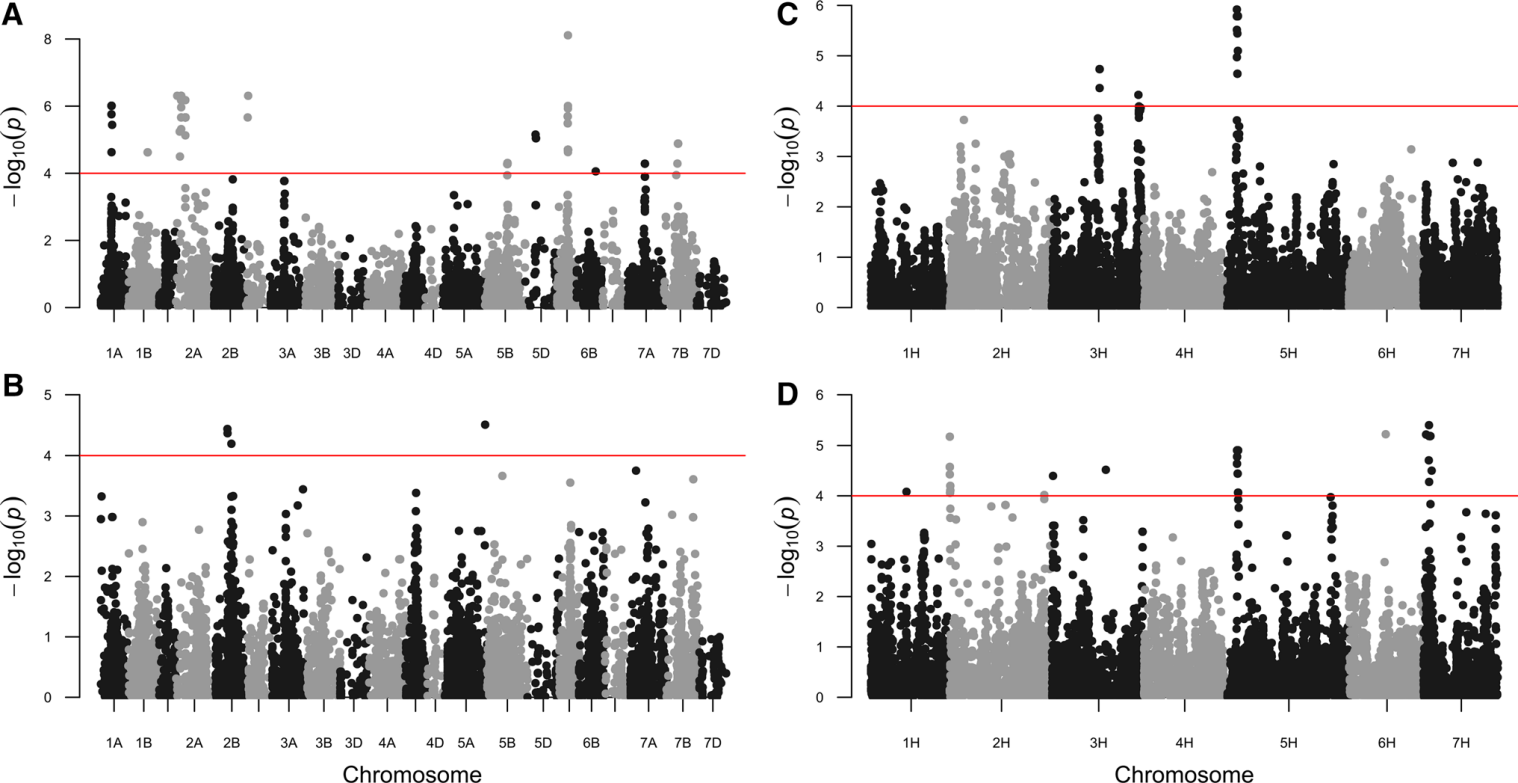
EnvGWAS for variety age. Manhattan plots of the four panels are shown. On the x-axis genetic positions based on the consensus map (Wang et al. 2014) are displayed for **a** UK winter wheat and **b** Brazilian spring wheat panels; for barley pseudo-genetic map positions that relate to the physical positions (Bayer et al. 2017) of the UK winter (**c**) and spring (**d**) barley panels are shown. On the *y*-axis − log_10_ (*p*)-values are displayed. The red line indicates the threshold value of the significance corresponding to − log_10_ (*p*) = 4

*EnvGWAS wheat*. Using variety age for EnvGWAS in the UK winter wheat panel (*n* = 404) identified thirteen significant (− log_10_ (*p*) > 4.0) genomic regions, of which four loci were found to be highly significant (− log_10_ (*p*) > 6.0), located on chromosomes 1A, 2A, 2D and 6A (Fig. 1a, Table 1**,** Supplementary Table S2). Subsequently, the region on 2D showed an identical genotyping profile to that of 2A (Supplementary Fig. S2) indicating errors in the genetic map of Wang et al. 2014, and we did not pursue the 2D region further. For example, the peak marker on 2D (BS00022799_51) correlates perfectly with nine markers on 2A that are also significantly associated such as (BS00080836_51 mapped on chromosome 2A at 158 cM). In Brazilian spring wheat (*n* = 355), three significant genetic loci were detected, two on chromosome 2B (251 cM, 318 cM) and one on 5A (710 cM), none of which were identified in the UK winter wheat panel (Fig. 1b, Table 1, Supplementary Table S2**)**.

**Table 1:** Summary of the significant hits detected by EnvGWAS on variety age

Pop name	SNP name	Chrom	Position (cM)	Ref-allele	Ref-Allele-Freq	− log(*p*)	Effects
Winter wheat	wsnp_Ex_c572_1138339	1A	221.0	A	0.50	6.01	− 7.61
	Kukri_c18109_682	1B	350.0	A	0.92	4.62	11.64
	Excalibur_c15379_1305	2A	20.0	A	0.66	6.31	9.50
	RFL_Contig4030_493	2A	162.0	A	0.65	5.24	8.32
	BS00071630_51	2A	87.0	A	0.66	6.18	9.26
	IACX6178	2A	158.0	A	0.66	6.18	9.26
	BS00022799_51	2D	33.0	A	0.66	6.31	9.50
	BobWhite_rep_c60245_107	5B	381.0	A	0.13	4.31	6.94
	BS00021901_51	5D	180.0	T	0.85	5.04	9.58
	BS00022120_51	6A	190.0	T	0.83	8.11	12.87
	Kukri_c16404_100	6B	322.0	A	0.06	4.06	10.33
	Kukri_c67076_479	7A	383.0	A	0.14	4.29	8.48
	BobWhite_c42974_184	7B	236.0	A	0.94	4.88	− 12.92
Spring wheat	Ku_c5725_892	2B	251.0	A	0.49	4.44	− 7.35
	RFL_Contig4849_702	2B	318.0	T	0.76	4.20	− 9.34
	RAC875_c8642_231	5A	710.0	A	0.08	4.51	− 13.21
Winter barley	JHI-Hv50k-2016-200,315	3H	68.7	A	0.29	4.74	− 1.95
	JHI-Hv50k-2016-222,233	3H	124.5	C	0.64	4.22	1.71
	JHI-Hv50k-2016-279,849	5H	19.2	A	0.73	5.92	− 1.87
Spring-barley	JHI-Hv50k-2016-37,011	1H	51.0	A	0.41	4.08	− 3.07
	SCRI_RS_148694	2H	0.0	A	0.42	5.17	− 2.59
	JHI-Hv50k-2016-149,544	3H	1.7	C	0.22	4.40	3.69
	JHI-Hv50k-2016-202,332	3H	77.7	C	0.95	4.52	-4.42
	JHI-Hv50k-2016-280,391	5H	20.5	C	0.12	4.90	3.38
	12_30230	6H	53.1	A	0.88	5.22	4.45
	JHI-Hv50k-2016-444,289	7H	7.8	A	0.93	5.40	5.37
Spring and winter barley	JHI-Hv50k-2016-58,537	2H	0.0	C	0.74	4.17	-2.15
	JHI-Hv50k-2016-71,264	2H	20.3	C	0.92	5.74	− 2.86
	JHI-Hv50k-2016-167,517	3H	45.2	C	0.92	4.15	3.07
	JHI-Hv50k-2016-200,365	3H	68.7	C	0.14	7.13	− 4.41
	JHI-Hv50k-2016-223,988	3H	126.6	C	0.80	4.29	3.64
	JHI-Hv50k-2016-279,907	5H	19.2	C	0.82	7.67	− 3.43
	JHI-Hv50k-2016-325,618	5H	105.0	A	0.09	4.51	3.53
	11_20546	5H	160.7	A	0.89	4.70	− 2.94
	JHI-Hv50k-2016-439,637	7H	3.8	C	0.05	5.56	− 4.65

Details in Supplementary Tables S2 and S3

*EnvGWAS Barley*. We identified three highly significant genetic loci in the winter barley panel (*n* = 297) and seven in the spring barley panel (*n* = 406) (Table 1; Fig. 1c, d); a summary of the associated markers is listed in Supplementary Table S3. Two significant loci were identified in both barley panels (chromosome 3H, ~ 68–70 cM; 5H, ~ 20 cM) (Fig. 1 and Supplementary Table S3). Subsequently, EnvGWAS was performed on the combined winter and spring panels (*n* = 704), identifying the same four major significant loci we identified in the spring panel alone (Supplementary Fig. S3a**,** Supplementary Table S3 and Table 1). In addition, we performed GWAS on seasonal growth habit itself (using winter and spring type as a trait), identifying three major genetic loci on the long arms of chromosomes 1H, 4H and 5H (Supplementary Fig. S3c), corresponding to major flowering time and vernalisation genes known to be the major determinants of winter and spring seasonal growth type (*PPD-H2* on chromosome 1H, *VRN-H2* on 4H and *VRN-H1* on 5H) (Cockram et al. 2007, 2015). EnvGWAS for variety age was then repeated with these QTL as covariates (Supplementary Fig. S3d). The most significant results mainly on chromosome 5H from the analyses with and without covariates changed little. However, the magnitude of other significant peaks differed, such as the locus on chromosome 1H.

### Validation of EnvGWAS based on trait analysis and a multi-founder experimental population

To validate the EnvGWAS analyses, we performed GWAS on the subset of 192 UK winter wheat varieties for which historical yield data were available together with EnvGWAS on variety age for direct comparison of the results. In this subset, we found that GWAS for yield identified the same genomic region on chromosome 1A (Supplementary Fig. S4a) as detected by EnvGWAS for variety age (Supplementary Fig. S4, Supplementary Table S2). This is the same region that we identified in EnvGWAS for variety age in the complete set of 404 UK wheat varieties. Interestingly, while the chromosome 5A QTL was detected with low significance (− log_10_ (*p*) = 4.45) by GWAS on yield, it was not identified using EnvGWAS on variety age. These two loci (1A and 5A) together explained 23.7% of the yield variation. In addition, EnvGWAS analysis of variety lifespan detected a locus on chromosome 1B that was not detected in any other of our analyses.

Similarly, EnvGWAS on variety age and GWAS on yield was repeated using the subset of 197 winter and spring barley varieties for which historical yield data were available, detecting highly significant hits (− log_10_ (*p*) > 4.0) on chromosome 5H for variety age, variety lifespan and yield, using seasonal growth habit as a covariate (Supplementary Fig. S5, Supplementary Table S3). It is noteworthy here that the analysis of our subset of 197 lines consistently identified a highly significant genetic locus on the short arm of chromosome 3H for variety age, variety lifespan and yield which was unidentified in the combined analysis of 703 varieties. In this case, however, another SNP (JHI-Hv50k-2016-151,847 “4.6 cM”) in a close location was close to significance (− log10*p* = 3.7) for variety age in the same region (Figs. S3a, S3b).

Together, the two loci (3H and 5H) explained 12.36% of the yield variation. An additional peak was detected with EnvGWAS for variety lifespan on the long arm of chromosome 2H.

To further validate our EnvGWAS findings, we analysed data from a 16 founder wheat multi-parent advanced generation inter cross (MAGIC) population consisting of 550 recombinant inbred lines generated by inter-crossing 16 wheat varieties released between 1935 and 2004 (Scott et al. 2021). We found that the three major genomic regions previously identified by EnvGWAS of variety age on chromosomes 1A, 2A and 6A were also significant in the MAGIC population for several yield and grain-related traits as well as for agronomic traits (Supplementary Table S4). Further details of the 213 agronomic and disease resistance traits analysed and the corresponding significance levels are listed in Supplementary Table S4.

### Allele shift over time

To illustrate the changes in allele frequency present in our variety collections over time, the allele carried (jittered) by each variety was plotted on the *Y*-axis against the age of the variety on the *X*-axis (Supplementary Fig. S6) for the major genomic regions identified by EnvGWAS on variety age (Supplementary Table S5–S8). In addition, graphical genotyping of all the significantly associated SNPs (–log_10_ (*p*-value) > 4.0) displays the allele changes over time (Supplementary Fig. S2). Different patterns and intensities of selection are evident across chromosomal regions over time. For wheat, these fall into three broad classes: (1) late introduction of ‘modern’ alleles followed by a rapid increase in frequency (Supplementary Fig. S6a), (2) retention of both ‘modern’ and ‘old’ alleles at similar frequency across time (e.g. Supplementary Fig. S6e), (3) relatively early introduction of the ‘modern’ allele, followed by its retention at low frequency (e.g. Supplementary Fig S6f). Details of the alleles-shift examples are provided in Supplementary Notes. In barley, the plots illustrated both gradual and rapid shifts in allele frequency at the genomic regions identified by EnvGWAS on variety age (Supplementary Fig. S6i-n). For example, for the UK spring barley genetic locus on chromosome 7H (~ 8.8 Mbp), only one allele was present until 1992 (Supplementary Fig. S6n and Supplementary Table S7), after which the ‘modern’ allele remained at low frequency, even among modern varieties. A genomic region on chromosome 5H, which was identified separately in winter and spring barley, displays a pattern where the ‘modern’ allele is introduced in 1986, after which both alleles are found at intermediate frequencies among the most recent varieties in winter barleys. However, modern spring barleys were predominantly of ‘modern’ allele type.

We accumulated the number of contemporary alleles carried by each variety at the significant loci for each of the four populations. Supplementary Fig. S7 visualises the joint cumulative change of allele frequencies over time for these significant regions. For UK winter wheat, only a handful of modern varieties carry all the modern alleles compared to Brazilian spring wheat where many more varieties carry all the detected modern alleles. However, more significant alleles were detected in the UK (12 compared to 3 in Brazil) so the probability of a variety carrying all modern alleles (though selection or sampling) is likely reduced. Interestingly in barley, the modern alleles were more dispersed among the modern varieties. It is still to be seen if there are major benefits in bringing together all these alleles in a single variety.

### EigenGWAS scans

While EnvGWAS allowed us to use variety age to investigate the genomic regions underlying QTL for yield and adaptation, we hypothesised that the complementary method, EigenGWAS, would allow us to detect changes in larger-scale structural variants in our target crop genomes over time. For instance, we detected the well documented 1B/1R translocation of wheat in the present study.

**Fig. 2:**
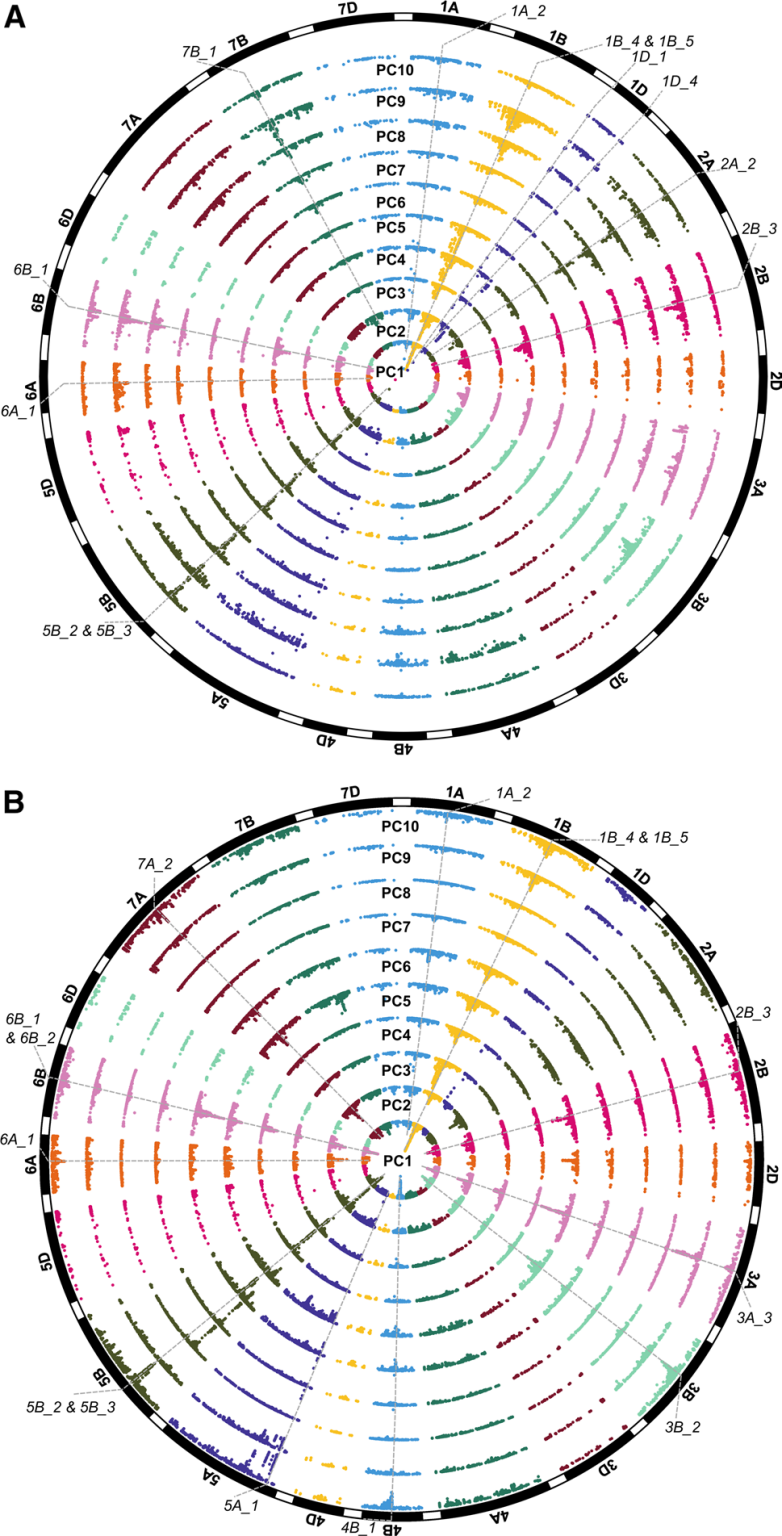
Wheat EigenGWAS for the first ten principal components (PCs). Circular plots of the two wheat panels investigated are shown. Genetic positions based on a consensus map (Wang et al. 2014) are displayed for **a** UK winter and **b** Brazilian spring wheat panels. Chromosomal introgressions significant across multiple PCs are highlighted (See Supplementary, Table S10)

After determining the first ten PCs in each of our UK and Brazilian wheat populations (Supplementary Table S9), EigenGWAS detected numerous significant hits (*N* = 11,567 SNPs with − log_10_ (*p*) > 4.0) (Fig. 2 & Supplementary Table S10). Since most of the variation among the panels (> 30%) is captured by the first ten PCs (only < 1.8% with PC10), we did not extend our analysis beyond these. Seven genetic loci distributed on chromosomes 1A, 1B, 2B, 5B, 6A and 6B were found to be significant for multiple PCs, in both the Brazilian (spring) and UK (winter) panels (Fig. 2). These loci corresponded to major chromosomal introgressions from related cereal species into wheat (Supplementary Table S10). For instance, the 1B locus co-locates with the chromosome 1B/1R introgression from rye (*Secale cereale*), which is known to regulate multiple traits including disease resistance and yield (Rajaram et al. 1983; Heslop-Harrison et al. 1990). We identified an additional seventeen putative introgression that were supported by a recent introgression survey by Cheng et al. 2019, along with another 58 novel putative introgressions (Supplementary Table S10). Among these novel putative introgressions were regions on chromosome 5B, depicted in Fig. 2 as 5B_2 and 5B_3, which displayed amongst the most significant hits across the UK and Brazilian wheat data sets and multiple PCs. Interestingly, two highly significant genomic regions (1A_2 and 5A_5) identified by EigenGWAS on PC2 in winter wheat were also detected by GWAS on yield in the validation data set (Supplementary Table S11). In addition, three genomic regions (5B_2, 6A_1 and 7B_1) identified in the winter wheat EigenGWAS analysis were also detected in EnvGWAS on variety age, suggesting that the approaches are not exclusively identifying different genomic regions (Supplementary Table S11). These introgression regions are not completely fixed in the modern varieties. For example 1B/1R and 5B_2 are still segregating (Supplementary Fig. S8), which is not surprising as often wheat breeders rely on several wild species introgressions to diversify their germplasm (Walkowiak et al. 2020). In addition, these introgressions may have favourable effects on some traits and be disadvantageous for others and are therefore less likely to be fixed by selection.

**Fig. 3:**
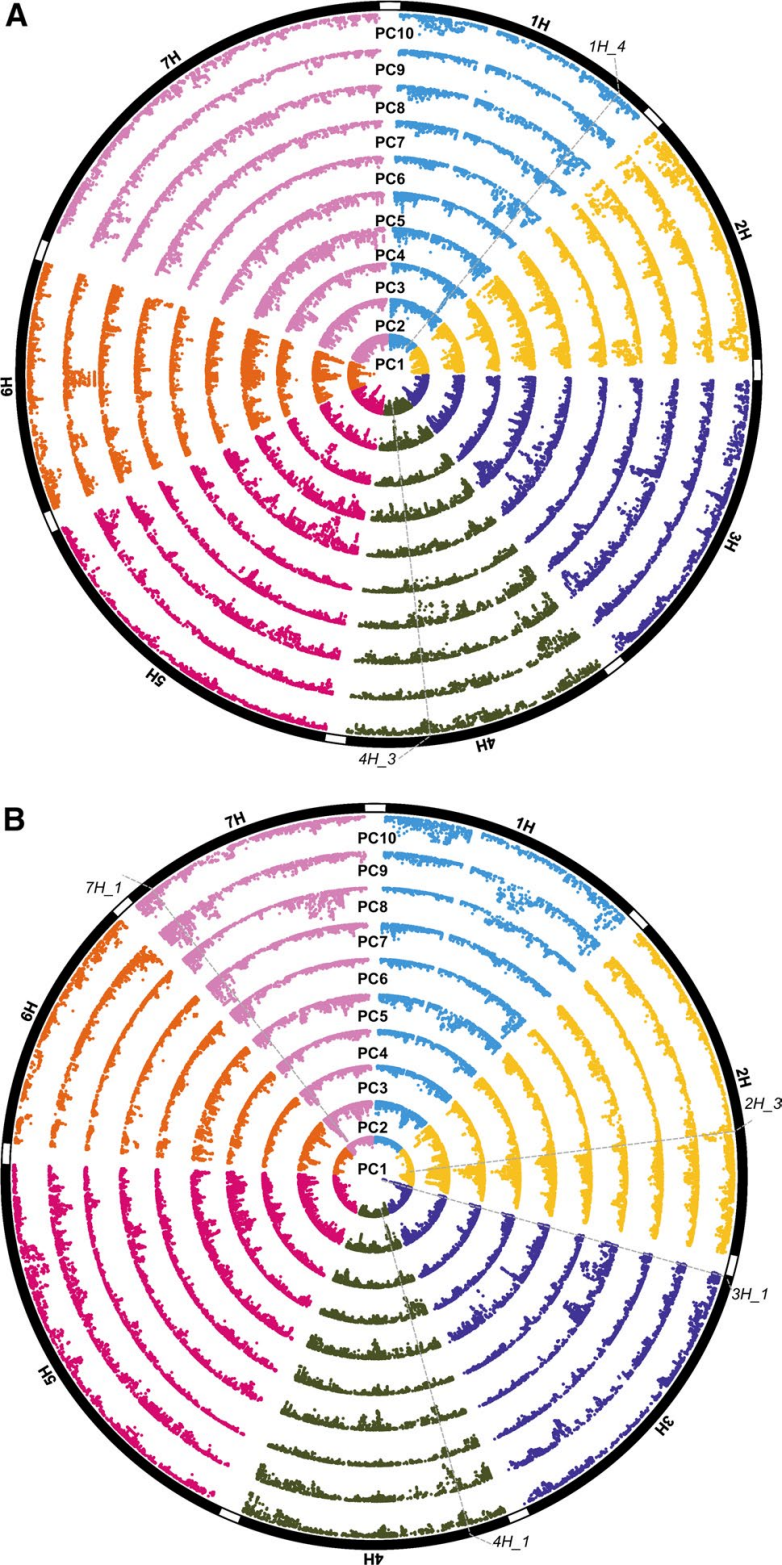
Barley EigenGWAS for the first ten principal components (PCs). Circular plots of the four panels are shown. Pseudo-genetic map positions that relate to the physical positions (Bayer et al. 2017) are displayed for **a** UK winter and **b** UK spring barley panels. Chromosomal introgressions significant across multiple PCs are highlighted (see Supplementary, Table S12)

In contrast to wheat, EigenGWAS in the winter and spring barley varieties did not detect any major loci with highly significant peaks across multiple PCs (Fig. 3 and Supplementary Tables. PCs’ variation in Table S9 & results in Table S12). However, two genomic regions in winter (1H_3 and 4H_3) and three in spring barleys (2H_3, 3H_1 and 7H_1) were identified in at least three PCs. Peaks were also identified close to the locations of known genes controlling flowering time and height (Supplementary Table S12), e.g. the PC5 hit on chromosome 3H ~ 632 Mbp (explaining 2.46% of the variation) is near the semi-dwarfing gene *sdw1* in spring barley. Interestingly, one of the most significant hits in the spring barley panel (*3H_1*, identified using PC1 and explaining 6.91% of the variation) was also detected using EnvGWAS on variety age and by GWAS on yield (Supplementary Table S13). Given the location of this hit in a highly recombinogenic region of the barley genome and that it was detected only in the spring barley panel, this may indicate a major locus under selection specific to spring barley breeding. No strong peak in winter barley was found for PC1, with the most significant peak obtained using PC6. As UK elite winter barley is more genetically diverse than UK elite spring barley, these results indicate that UK elite winter barley may be subjected to weaker selection pressures. Interestingly, hits on genomic regions (*5H_2* and *7H_1*) from the spring barley EigenGWAS analysis were also identified in GWAS analysis of seasonal growth-habit and variety age, highlighting the importance of these loci under selection (Supplementary Table 13).

## Discussion

We demonstrate that use of variety age for EnvGWAS can detect regions of crop genomes under selection during breeding. In addition, we show variety age is a good proxy for yield, with the genetic loci identified for wheat validated in an independent experimental multi-founder population (Scott et al. 2021). Lastly, we showed that the genetic loci detected by EnvGWAS showed gradual, as well as sharp, shifts in allele frequency over time, indicating subtle changes at these loci by breeders, which are less discernible to detection using approaches such as partitioning the populations on age and searching for differences based on Fst. We illustrate a dynamic change in alleles at specific loci over time through the deployment of plots that capture different patterns of selection in both wheat and barley that are easily discernible. However, the use of age as a surrogate for yield and other traits under selection is not perfect; for example, we failed to detect a 3H locus for variety age in the combined analysis of 703 barley varieties although it was highly significant for variety age, variety lifespan and yield in the subset of 197 lines. It is possible, but unproven, that the inclusion of the kinship matrix to reduce the frequency of false positives overcorrects when applied to historical datasets spanning long time intervals. In agreement with this, the full set of lines span the years 1963–2016, whereas the subset spans years 1964–2005, and a simple linear regression of age of variety on the 3H SNP [JHI-Hv50k-2016-150851] gives a − log10(*p*) of 14.0 compared to − log10(*p*) of 7.2 in the phenotyped subset.

It is perhaps not surprising that selection of loci varies between the UK winter and Brazilian spring wheat, given that the target agricultural environments and growth types are very different. Wheat yields in both Brazil and the UK have improved greatly over the years (Rodrigues et al. 2007; Mackay et al. 2011). Our contrasting results in wheat indicate that different sets of genes have been selected over the years and are likely involved in both yield component and local adaptation traits. Future efforts will shed more light on the types of genes underpinning these loci, allowing changes in allelic diversity over the years to be investigated.

Our results for UK barley contrast with those for UK wheat. Firstly, more hits were associated with variety age in spring compared to winter barley, and secondly an identical peak on chromosome 5H (at ~ 19 cM, ~ 7.5 Mbp) was identified in both panels (as well as in the combined spring and winter analysis). This is surprising as breeders rarely cross spring and winter barley, and since the breeding targets in the two pools differ (malting and largely animal feed, respectively). To further investigate this region, we tested the candidate SNPs against phenotypic data available from national trial data (Supplementary Table S14), finding it to be associated with several malting quality traits, powdery mildew resistance and yield in fungicide-untreated trials. These findings suggest the potential importance of this region for breeding for disease resistance and end-use quality. Interestingly, this region on 5H houses a cluster of terpene synthases that have been implicated in fungal disease resistance in other species (Chen et al. 2020) and that potentially have been selected alongside direct targets such as *Mla* and *mlo* genes (Jorgensen 1992).

The detection of significant hits with EnvGWAS provides an opportunity to explore their relationship with yield and other agronomically important traits. Some hits coincide with previously published QTL in wheat and barley, for example the highly significant loci on wheat chromosomes 1A and 6A (Zanke et al. 2015; Lehnert et al. 2018; Yang et al. 2020). Our EnvGWAS hits on chromosomes 1A and 2A also overlapped with the reduced diversity peaks identified in the recent analysis of the UK wheat pedigree by Fradgley et al. 2019. Specifically, the 2A locus may correspond to a stripe rust resistance gene described by Beukert et al. 2020, as the peak markers overlap. Interestingly, a group of R genes *Lr37*-*Yr17*-*Sr38* (Helguera et al. 2003) which were important sources of resistance in the past also lie in this region and might be more plausible candidates, rising in frequency before their resistance broke down. Similarly, the highly significant genetic locus on the short arm of barley chromosome 3H for variety age and yield found in the subset of 197 barley lines corresponds to the genomic region associated with a malting quality trait, hot water extract, in UK spring barley that demonstrated a major change in allele frequency over the last thirty years (Looseley et al. 2020). In addition, the region identified on chromosome 3H (~ 68 cM) for variety age in winter barley in the larger dataset has been shown previously to be associated with yield component traits (grain length and grain area) in European winter barley (Xu et al. 2018).

Similarly, in barley, the region identified on chromosome 2H (~ 65 cM, ~ 621 Mbp) for variety lifespan has been shown previously to be associated with yield and yield component traits (Sharma et al. 2018; Xu et al. 2018) and may correspond to the *OsBR1*/*D61* candidate genes reported previously that are associated with yield traits in barley (Sharma et al. 2018; Xu et al. 2018). Such a correspondence could be due to promising varieties being under cultivation for longer as they harboured a yield advantage over the varieties cultivated for a shorter period.

This is interesting as old varieties, despite being less productive than modern varieties, were under cultivation for longer periods. It may, however, be noted that with the introduction of modern breeding practices yield has increased, but with drastic effects on variety lifespan due to the more frequent introduction of new varieties that outperform contemporary varieties. In wheat, EnvGWAS on variety lifespan also identified a hit on chromosome 1A that co-located with a hit for variety age. This further indicates a direct relationship between variety age and variety lifespan in wheat and barley.

Using EigenGWAS, we detected major introgressions in the wheat varietal panels investigated, with several of these found to be in common between the UK winter and Brazilian spring wheat panels, indicating their wide use in breeding. Scott et al. 2021, analysing the 16 founder MAGIC population we used in our validation studies, proposed a major role for multiple introgressions from wild species in UK wheat breeding to date. In contrast, EigenGWAS results in barley provide no evidence of a similar pattern of introgressions in either the winter or spring panels. Wheat and barley breeding differ in their exploitation of genetic resources. In wheat, several alien-introgressions from related species are known to have occurred (Gill et al. 2011). While wheat is an allohexaploid and can support large tracts of non-recombining alien chromosome, this may not be the case in diploid barley. However, examples of introgressions in barley from landraces and spontaneous mutant lines for agronomically important genes have been reported, such as the semi-dwarfing allele *sdw1d* from the variety Diamant and the disease resistance gene *mlo11* from Ethiopian landraces (Haahr and Wettstein, 1976; Jorgensen 1992).

Interestingly, within the genomic region of 6A_1, detected by EigenGWAS in wheat (a non-recombining peri-centromeric region) lies the gene *TaGW2* (Zhang et al. 2018) which influences grain-weight and protein content traits that further suggest that the present approach is very effective in discovering genomic regions undergoing selection for yield. Another interesting finding is that the semi-dwarfing *Rht2* gene in wheat (chromosome 4D) was not detected despite its importance in the breeding history of the crop. This could be due to population structure control of the analyses. In the case of *Rht2*, it is noteworthy that GWAS on a panel of French, German and UK lines failed to detect an effect on yield or height unless a locus-specific marker was used (Bentley et al. 2014; Ladejobi et al. 2019), suggesting weak LD and low marker coverage on the 4D chromosome as the cause of failure here too.

## Conclusion

Breeding has resulted in considerable and sustained genetic improvement of wheat and barley in recent decades, and our results identify at least some of the major loci that have contributed, and are still contributing, to these improvements. Using EnvGWAS, we demonstrate the utility of analysing variety age as a surrogate for traits selected by breeders to detect the genetic loci under selection over time and to assess the temporal changes in their respective allele frequencies. For UK cereals, trends over time suggest that these loci are likely QTL for yield or yield components. While the resolution of this study in the non-recombining peri-centromeric regions is insufficient to definitively associate known QTLs with the loci we have found, several such QTLs were found. EigenGWAS on the same data proved a simple method of detecting contrasting features of genome organisation in wheat and barley, and in some cases these too could be related to traits. We advocate the use of variety age as a surrogate trait and the use of EnvGWAS and EigenGWAS to identify the genetic loci under selection that have underpinned the productivity gains made via breeding. These extensions to GWAS that exploit historical datasets are useful additions to the analysis toolbox of crop quantitative genetics.

## Supplementary Information

Below is the link to the electronic supplementary material.

Supplementary file1 (DOCX 1509 KB)

Supplementary file2 (XLSX 65 KB)

Supplementary file3 (XLSX 30 KB)

Supplementary file4 (XLSX 82 KB)

Supplementary file5 (XLSX 18 KB)

Supplementary file6 (XLSX 269 KB)

Supplementary file7 (XLSX 11 KB)

Supplementary file8 (XLSX 1003 KB)

Supplementary file9 (XLSX 24 KB)

Supplementary file10 (XLSX 255 KB)

Supplementary file11 (XLSX 12 KB)

Supplementary file12 (XLSX 17 KB)

## Data Availability

Genotypic data sets of the study are available from NIAB, UCL and JHI via following websites: https://www.niab.com/research/agricultural-crop-research/resources. http://mtweb.cs.ucl.ac.uk/mus/www/MAGICdiverse/index.html. http://www.barleyhub.org/projects/impromalt/.
