# Supplementary material for: Trends of genetic changes uncovered by Env- and Eigen-GWAS in wheat and barley
Source: Theor Appl Genet. 2021 Nov 15;135(2):667–78. doi: 10.1007/s00122-021-03991-z (PMC8866380; doi:10.1007/s00122-021-03991-z)
Supplement: Supplementary file 1 — Supplementary file1 (DOCX 1509 KB) [file 122_2021_3991_MOESM1_ESM.docx]

**Supplementary Information**

**Trends of genetic changes uncovered by Env- and Eigen-** **GWAS in wheat and barley**

Rajiv Sharma^a^, James Cockram^b^, Keith A. Gardner^b^, Joanne Russell^c^, Luke Ramsay^c^, [William TB Thomas](javascript:;)^c^, Donal M. O’Sullivan^d^, Wayne Powell^a^, Ian J. Mackay^a1^

Ian J. Mackay

Email: ian.mackay@sruc.ac.uk

**This file includes:**

Supplementary text

Figures S1 to S5

Tables S1 to S14

SI References

**Other supplementary materials for this manuscript include the following:**

Datasets S1 to S14

**Supplementary Information Text**

**Supplementary Results**

**Detail examples from allele-shift over-time**

Trends from the wheat Eigen-GWAS analysis are provided in Supplementary Fig S6. For example, in the UK wheat panel chromosome 1A region, the ‘old’ allele was present until 1960 when the cultivar Maris Widgeon was released. From 1985 onwards, varieties mostly carried the ‘modern’ allele although a few recent cultivars like Stigg (released 2007) and Panorama (2009) still retain the ‘old’ allele (Supplementary Fig. S6a). Notably, identical patterns were detected on chromosome 2A (~87 cM & ~158 cM (Supplementary Fig. S6b-c). However, as these markers were found to be in high LD (r=-0.99, p<0.01) and their physical map positions are close (14.8 and 15.6 Mbp, chromosome 2A), it is likely that the genetic positions are wrong. The variety Eclipse introduced in 1950 was the first that carried the ‘modern’ alleles at the 2A locus, with ‘old’ alleles persisting at low-frequency even among the modern cultivars. Brazilian-spring wheat show similar trends, although with gradual changes rather than drastic changes in allele frequency (Supplementary Fig. S6e-g). However, a marker (SNP: wsnp_Ex_rep_c66763_65120251) un-unassigned on the consensus genetic map used (1) but placed on physical map chromosome 3A (331618247 bp) displayed low-frequency throughout the years even in modern cultivars (Supplementary Fig. S6h).


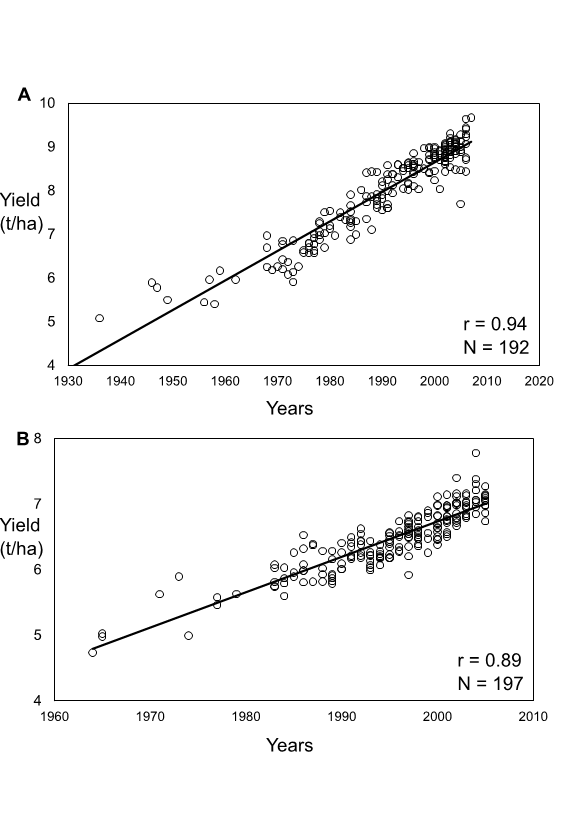


**Fig. S1** Linear trend in variety year of release and yield (t/ha). (a) UK wheat; (b) UK barley.

**Fig. S2** Graphical genotyping plots of significant regions (–log_10_(*p*-value) > 4.0) displaying allele frequencies change over-time in wheat (UK-wheat: a & Brazilian-wheat: b) and barley panels (winter: c & spring: d). Varieties, in rows, are in order of ascending age. Green = allele first observed in the panel and Red = allele observed subsequently. For comparison, for each panel, a small number of non-significant unlinked SNPs are also shown, labelled ‘NS’). Plots produced using Flapjack (Milne et al. 2010).

**
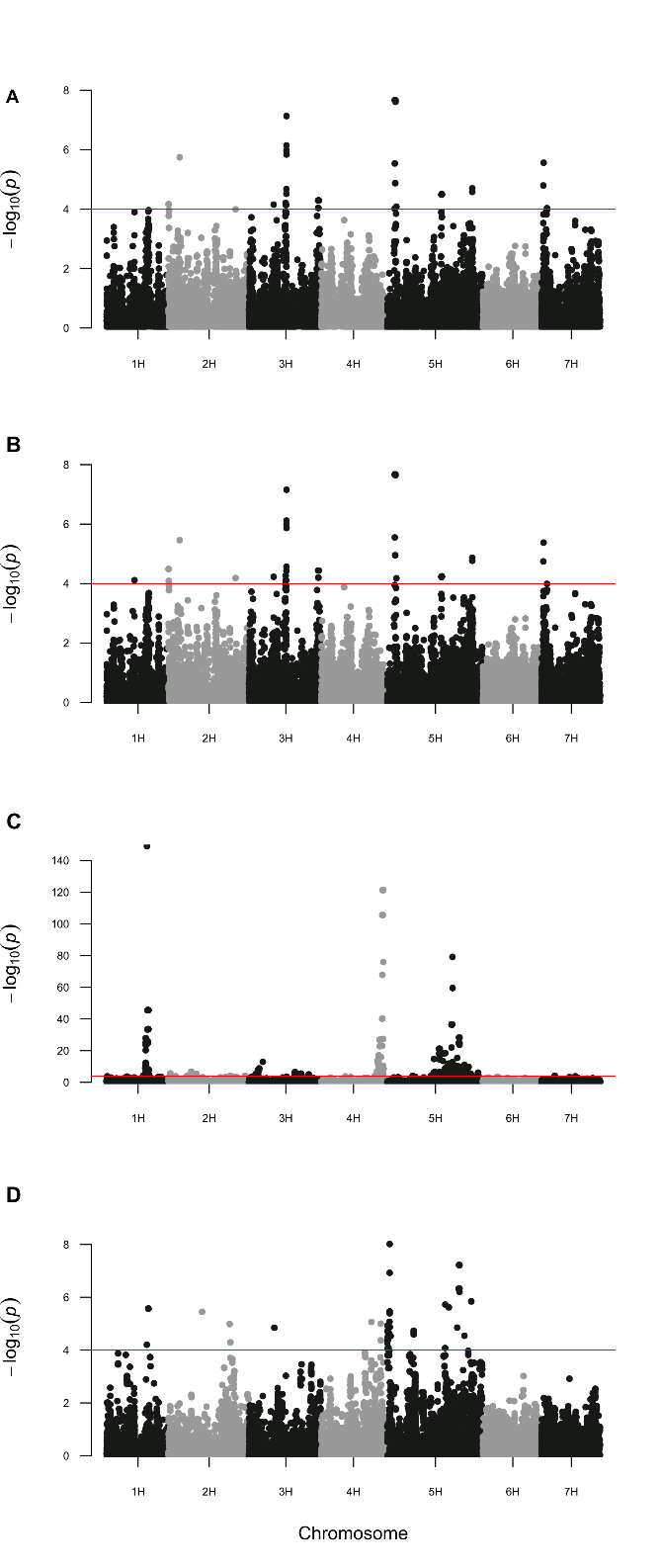
**

**Fig. S3** UK barley Manhattan plots for EnvGWAS from combined winter and spring varieties (*n*=704). (A) Manhattan plot for the age from the analysed barley varieties; (B) analysis using seasonal growth-habit (winter and spring-type) as a covariate; (C); GWAS analysis of seasonal growth-habit; (D) GWAS analysis of age with the four peaks identified in C as covariates.

**
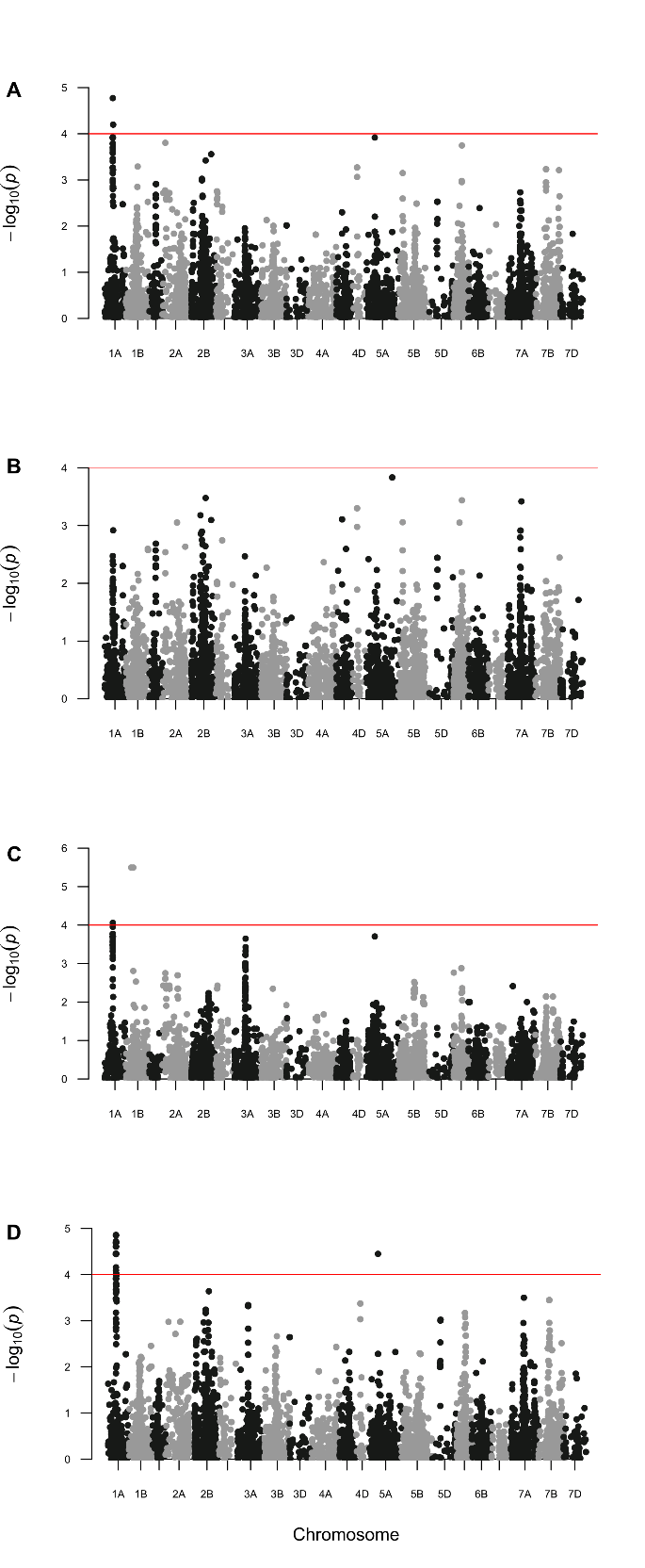
**

**Fig. S4** UK winter wheat Manhattan plots for EnvGWAS validation using a subset of 192 varieties. Manhattan plots are shown for: (A) the first year of variety in national trials; (B) the last year each variety is tested in national trials; (C) variety life-span, i.e. duration of a variety in trial; (D) GWAS on yield.

**
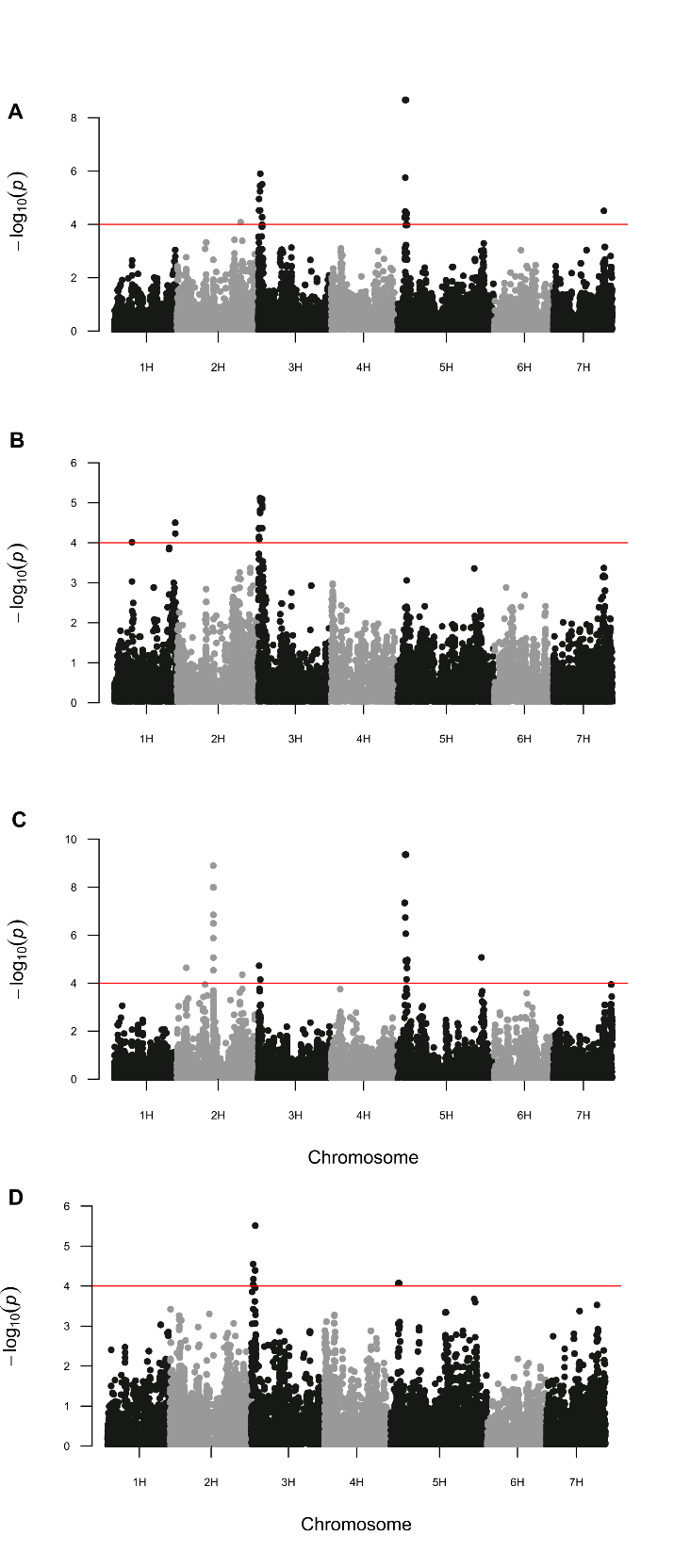
**

**Fig. S5** UK barley Manhattan plots for EnvGWAS validation using a subset of 197 varieties. Manhattan plots are shown for: (A) the first year of variety in National trial; (B) the last year each variety is tested in national trials; (C) variety life-span, i.e. duration of a variety in trial; (D) GWAS on yield.

**
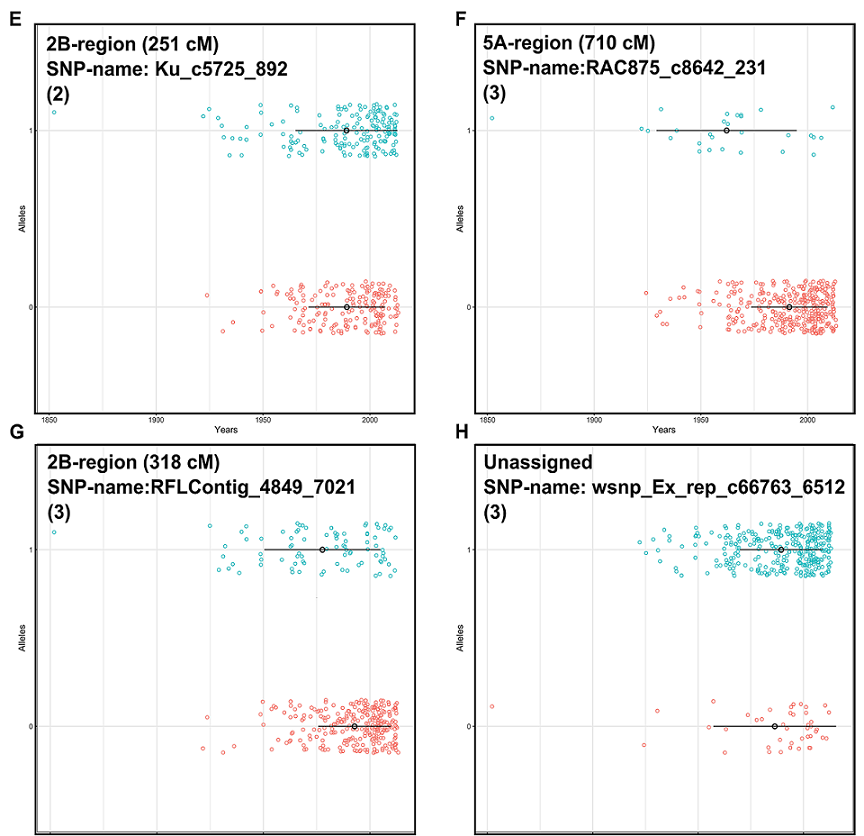
**

**Fig. S6** Plots of temporal changes in allele frequencies. Each variety is plotted against its year of entry into trial with colours distinguishing the allele carried by that variety: UK wheat A-D, Brazilian-wheat E-H, winter barley I-J & spring barley K-N. For wheat, the numbers 1-3 in parenthesis within the panel categorize the allele changes over time as (1) rapid increase in frequency in modern varieties; (2) similar frequency of old and young alleles in modern varieties; and (3) the ‘modern' allele was introduced very early and retained at a low frequency. Horizontal black lines and points delineate the 0.25. 0.5 and 0.75 quantiles of variety age for each allele. Allele codes are based on the data available on the NIAB and IMPROMALT websites.


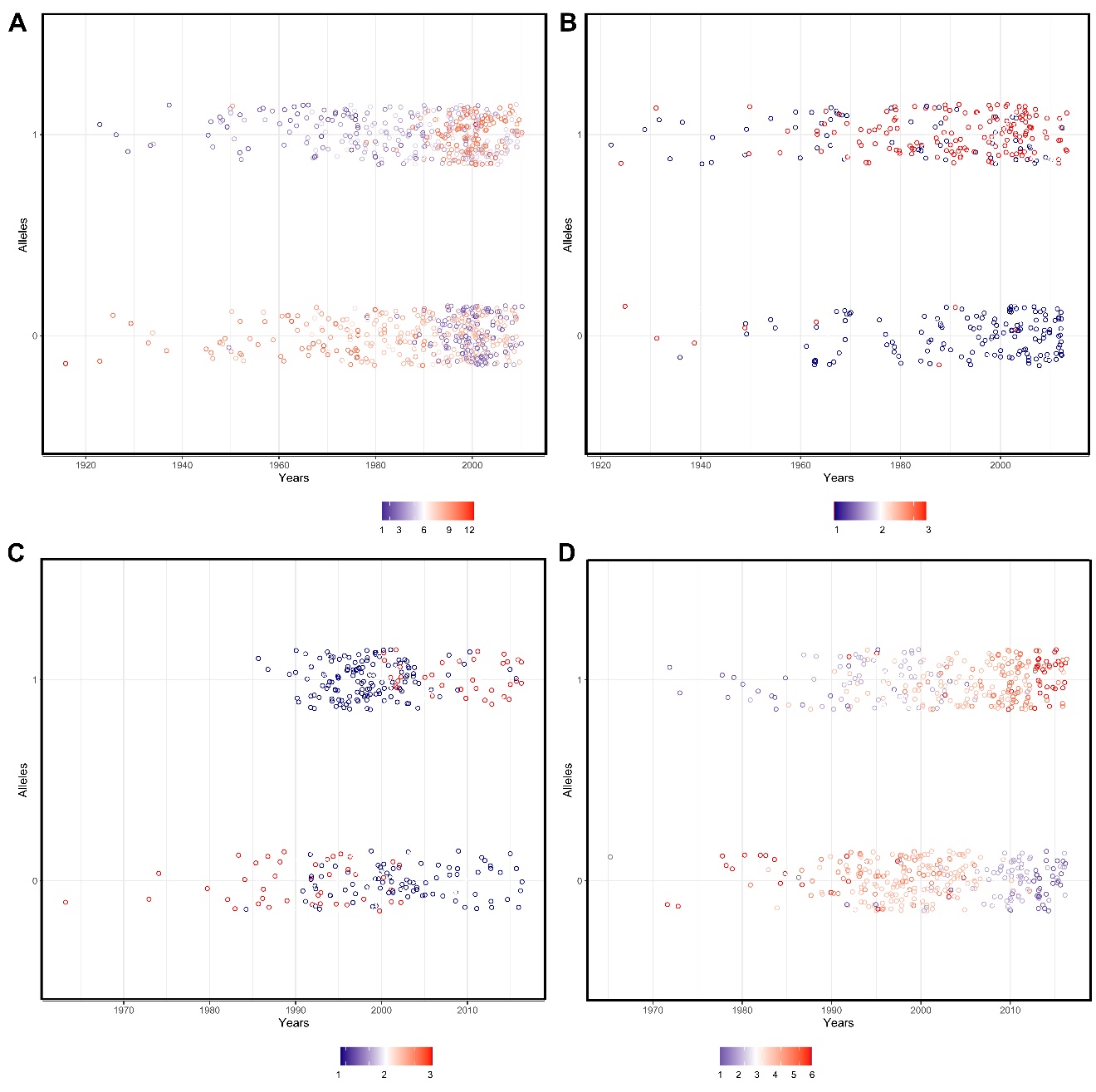


**Fig. S7** Varieties plotted against age to display cumulative change in allele frequencies over time at significant loci. A: UK winter wheat, B: Brazilian spring wheat, C: winter barley, D: spring barley. The number of age-decreasing (i.e. modern) alleles carried by each variety is differentially coloured with the key given in the bottom of each panel. Old alleles are scored 0 and modern alleles 1.

**B**


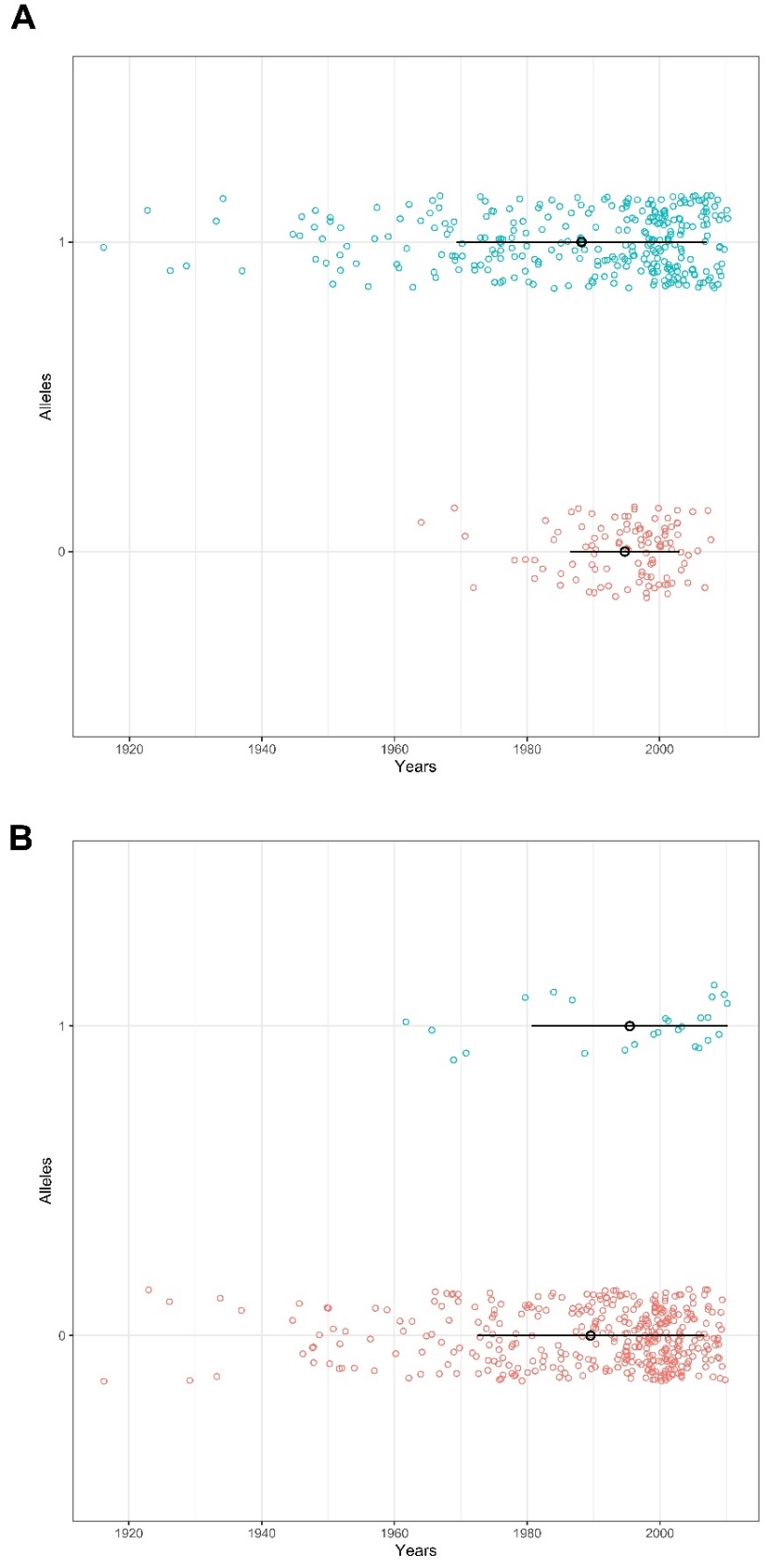


**Fig. S8** Plot of two major EigenGWAS loci displaying temporal changes in allele frequencies in winter wheat. A: 1B/1R-region; B: 5B_2-region. Varieties with 1B/1R introgression are represented by allele 0, whereas those with 5B-2 introgression are represented by allele 1.

**Supplementary Tables**

**Table S1**. Overview of the cultivars and the year of release. Also, provided last-year of registration when available.

**Table S2**. Summary of the marker-trait associations in wheat panels.

**Table S3**. Summary of the marker-trait associations in barley panels.

**Table S4**. Summary of the MAGIC-wheat analysis corresponding to three major genomic regions that are significant in Env-GWAS of age.

**Table S5**. Overview of the alleles-shifts over time for EnvGWAS peak regions of the UK winter wheat panel. Significant SNP marker names from EnvGWAS are given with variety names and the year of release.

**Table S6**. Overview of the alleles-shifts over time for EnvGWAS peak regions of the Brazilian wheat panel.

**Table S7**. Overview of the alleles-shifts over time for EnvGWAS peak regions of the UK winter barley panel.

**Table S8**. Overview of the alleles-shifts over time for EnvGWAS peak regions of the UK spring barley panel.

**Table S9**. Percent variation explained by first ten principal components (PC1-PC10) from the four populations are shown.

**Table S10**. Summary of the significant GWAS hits using ten PCAs in wheat. Where applicable, introgression regions are numbered following ^a^Cheng et al. 2019 and ^b^Scott et al. 2020.

**Table S11**: Overview of the peaks identified across envGWAS and eigenGWAS analysis from wheat.

**Table S12**. Summary of the significant GWAS hits using ten PCAs in barley.

**Table S13**. Overview of the peaks identified across EnvGWAS and EigenGWAS analysis from barley.

**Table S14**. Summary of the significant traits from the 5H-genomic region.

**SI References**

H. Cheng, *et al.*, Frequent intra- and inter-species introgression shapes the landscape of genetic variation in bread wheat. *Genome Biology* **20**, 1–16 (2019).

I. Milne, et al., [Flapjack – graphical genotype visualization.](http://bioinformatics.oxfordjournals.org/content/26/24/3133)*Bioinformatics* **26**, 3133-3134 (2010).

M. F. Scott, *et al.*, Limited haplotype diversity underlies polygenic trait architecture across 70 years of wheat breeding. *Genome biology*, **22**, 1-30 (2021)

S. Wang, et al., Characterization of polyploid wheat genomic diversity using a high-density 90 000 single nucleotide polymorphism array. *Plant Biotechnology Journal* (2014) https:/doi.org/10.1111/pbi.12183
